# USP14 is a predictor of recurrence in endometrial cancer and a molecular target for endometrial cancer treatment

**DOI:** 10.18632/oncotarget.8821

**Published:** 2016-04-18

**Authors:** Rachel Isaksson Vogel, Tanya Pulver, Wiebke Heilmann, Ashley Mooneyham, Sally Mullany, Xianda Zhao, Maryam Shahi, James Richter, Molly Klein, Liqiang Chen, Rui Ding, Gottfried Konecny, Stefan Kommoss, Boris Winterhoff, Rahel Ghebre, Martina Bazzaro

**Affiliations:** ^1^ Masonic Cancer Center and Department of Obstetrics, Gynecology and Women's Health, University of Minnesota, Minneapolis, MN, USA; ^2^ Department of Women's Health, University Hospital Tuebingen University, Tuebingen, Germany; ^3^ Division of Basic and Translational Research, Department of Surgery, University of Minnesota, Minneapolis, MN, USA; ^4^ Department of Pathology and Laboratory Medicine, University of Minnesota, Minneapolis, MN, USA; ^5^ Center for Drug Design, Academic Health Center, University of Minnesota, Minneapolis, MN, USA; ^6^ Gynecologic Oncology, Hematology & Oncology Department, UCLA Medical Center, Santa Monica, CA, USA

**Keywords:** endometrial cancer, VLX1570, recurrence, biomarker, USP14

## Abstract

Endometrial adenocarcinoma is the most common gynecologic malignancy in the United States. Most endometrial cancer cases are diagnosed at an early stage and have good prognosis. Unfortunately a subset of patients with early stage and low grade disease experience recurrence for reasons that remain unclear. Recurrence is often accompanied by chemoresistance and high mortality.

Deubiquitinating enzymes (DUBs) are key components of the ubiquitin-dependent protein degradation pathway and act as master regulators in a number of metabolic processes including cell growth, differentiation, and apoptosis. DUBs have been shown to be upregulated in a number of human cancers and their aberrant activity has been linked to cancer progression, initiation and onset of chemoresistance. Thus, selective inhibition of DUBs has been proposed as a targeted therapy for cancer treatment.

This study suggests the DUB USP14 as a promising biomarker for stratifying endometrial cancer patients at diagnosis based on their risk of recurrence. Further USP14 is expressed along with the marker of proliferation Ki67 in endometrial cancer cells *in situ.* Lastly, pharmacological targeting of USP14 with the FDA approved small-molecule inhibitor VLX1570, decreases cell viability in chemotherapy resistant endometrial cancer cells with a mechanism consistent with cell cycle arrest and caspase-3 mediated apoptosis.

## INTRODUCTION

Endometrial adenocarcinoma is the most common gynecologic malignancy in the United States with an estimated 55,000 new cases in 2015 [1]. The majority of patients are diagnosed at an early stage with an overall favorable prognosis, although approximately 20% will die from the disease [1, 2]. Clinical factors such as grade, histology and surgical stage are important determinants of prognosis for endometrial adenocarcinoma. Among women with early stage and low grade endometrial cancer, representing 60% of endometrial cancers, 5-year overall survival is greater than 93% and adjuvant therapy beyond surgical hysterectomy offers no survival benefit [3–5]. A major and continued challenge in endometrial cancer management is the development of targeted therapies for those with early stage endometrial cancer at higher risk of recurrence and de-escalation of management for those at lowest risk.

The current risk stratification system relies heavily on histologic features, classifying women with endometrial adenocarcinoma into two groups, Type I or Type II [6]. Type I endometrial adenocarcinomas, the most common subtype, typically occur in the setting of excessive estrogen and consist of low grade endometrioid, hormone receptor positive cancers with good prognosis. Type II endometrial adenocarcinomas include non-endometrioid, high grade, TP53-mutated, hormone-receptor negative cancer and are associated with poor prognosis [7, 8]. Unfortunately, these risk groups fail to predict recurrence in some women as 15% of women with endometrioid endometrial cancer (Type 1) will experience recurrence and risk stratification based on histology or grade alone may fail to capture this subset of women [3, 9]. Therefore molecular markers are needed to facilitate risk stratification and treatment of early stage endometrial cancer.

The Ubiquitin-Specific Protease 14 (USP14) is a proteasome-associated deubiquitinating enzyme (DUB) responsible for cleaving ubiquitin chains from proteins destined for proteasome degradation. Aberrant expression of USP14 has been implicated in a variety of cancers, including multiple myeloma, colorectal cancer, lung cancer, and epithelial ovarian cancer [10–14]. Notably, aberrant expression of USP14 in epithelial ovarian cancer has been associated with poor prognosis [12]. Furthermore, pharmacological inhibition of USP14 with the FDA approved small-molecule inhibitor VLX1570 has been suggested as an alternative treatment method for cancer in a number of cancer settings, including breast and ovarian cancer [15, 16].

To date, the role of USP14 as a biomarker and molecular target in the endometrial cancer setting is largely unknown. In this study, we show that higher expression levels of USP14 are independently associated with recurrence in a retrospective cohort of women with stage I endometrial adenocarcinoma. Specifically our data indicate that after taking into account other known risk factors for recurrence, namely disease grade, histology, stage, receipt of adjuvant therapy and presence of lymphovascular space invasion, USP14 can be used as a biomarker to stratify stage I endometrial cancer patients according to risk of recurrence. Furthermore, we show that pharmacological inhibition of USP14 severely affects the viability of carboplatin resistant endometrial cancer cells with a mechanism consistent with arrest of the cells in the G2/M phase of the cell cycle followed by caspase-3 mediated onset of apoptosis. In light of these findings, we suggest that USP14 is a novel potential biomarker of recurrence in endometrial cancer, as well as a molecular target for its treatment.

## RESULTS

### Overexpression of USP14 is associated with recurrence in endometrial cancer

Measures of disease aggressiveness (stage, histology, and myometrial invasion) are known to be related to recurrence among women with stage I endometrial cancer, however a subset of patients with anticipated good prognostic factors will recur. We sought to determine whether USP14 expression level could serve as an independent marker of recurrence. To that end, the association between USP14 and recurrence in women with endometrial adenocarcinoma was evaluated using a retrospective cohort of stage I endometrial adenocarcinoma cases treated at our institution. A total of 107 patients with at least 36 months of follow-up, oversampling those who recurred, were included in the analysis. Comparisons indicated no differences in demographic and clinical factors between patients included and not included in the analysis.

Patients were on average 60.6±9.6 years old at diagnosis, most were white, obese, menopausal, and hypertensive. The relationships between demographic and clinical variables and recurrence within 36 months of diagnosis were explored to both describe the population and identify potential confounding factors. As expected, numerous clinical factors were statistically significantly associated with recurrence in this population, including disease stage IB, myometrial invasion >50%, tumor size ≥2 cm, and presence of lymphovascular space invasion (Table 1).

**Table 1:** Factors associated with endometrial adenocarcinoma recurrence within 36 months

	Did Not Recur (N=88)		Recurred (N=19)		
	N	%	N	%	p-value
**Age at Diagnosis**					0.92
<50 years	9	10.2	1	5.3	
50-69 years	66	75.0	15	79.0	
70+ years	13	14.8	3	15.8	
**Race**					0.42
Black	4	4.6	0	0.0	
White	72	81.8	19	100.0	
Other	8	4.6	0	0.0	
Unknown/Declined	4	9.1	0	0.0	
**Obese**					0.79
No	28	32.6	5	26.3	
Yes	58	67.4	14	73.7	
**Nulliparous**					0.35
No	59	73.8	13	86.7	
Yes	21	26.3	2	13.3	
**Menopausal**					0.73
No	15	17.4	2	11.1	
Yes	71	82.6	16	88.9	
**Hypertension**					0.71
No	33	37.5	8	42.1	
Yes	55	62.5	11	57.9	
**Diabetes**					0.96
No	69	78.4	15	79.0	
Yes	19	21.6	4	21.1	
**Histology**					0.31
Endometrioid	75	85.2	14	73.7	
Other	13	14.8	5	26.3	
**Squamous Component**					0.90
No	56	65.1	12	66.7	
Yes	30	34.9	6	33.3	
**Grade**					0.11
1	36	40.9	4	21.1	
2	30	34.1	6	31.6	
3	22	25.0	9	47.4	
**Disease Stage**					<0.0001
IA	75	85.2	8	42.1	
IB	13	14.8	11	57.9	
**Myometrial Invasion**					0.006
None	21	23.9	3	15.8	
<50%	54	61.4	5	26.3	
≥50%	13	14.8	11	57.9	
**Tumor Size**					0.07
≤2 cm	24	28.2	1	5.6	
>2 cm	61	71.8	17	94.4	
**Lymphovascular Space Invasion**					0.008
No	73	84.9	11	57.9	
Yes	13	15.1	8	42.1	
**Pelvic and Aortic Lymphadenectomy**					1.00
No	13	14.8	3	16.7	
Pelvic only	5	5.7	1	5.6	
Yes - both	70	79.6	14	77.8	
**Received Adjuvant Therapy**					0.24
No	59	67.0	10	52.6	
Yes	29	33.0	9	47.4	

**Figure 1:**
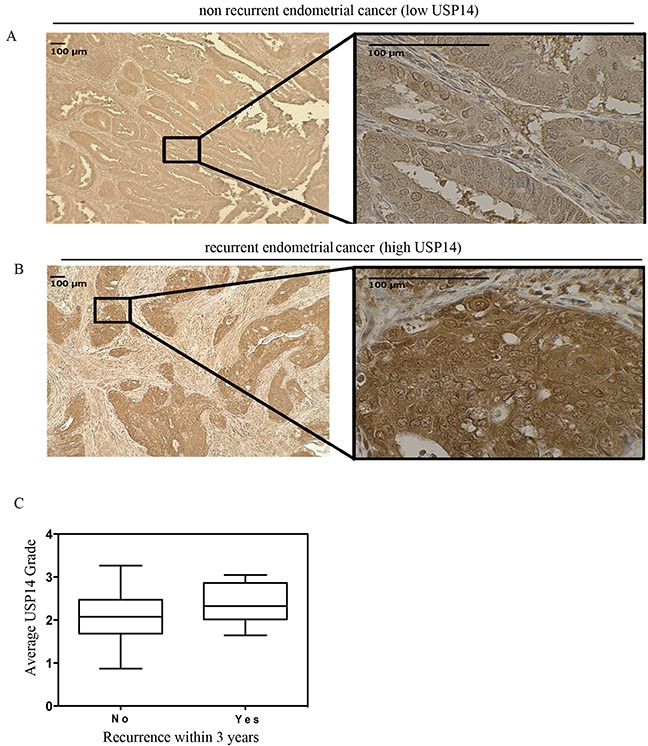
USP14 is overexpressed at diagnosis of endometrial adenocarcinoma among patients who eventually recur Immunohistochemical staining of USP14 in endometrial cancer tumors. USP14 is present in both the cytoplasm and the nucleus. Representative example patient with endometrial cancer with **A.** weak staining in a patient who did not recur and **B.** intense staining in a patient who did recur within 36 months. **C.** Boxplots depicting the average USP14 staining intensity at diagnosis for patients with endometrial adenocarcinoma who did not recur (left) and those who recurred (right) within 36 months of diagnosis. The median USP14 staining intensity was higher among those who recurred (2.3 vs. 2.0; p=0.02). Symbols on the boxplot are as follows: Box = 1st to 3rd (Q1-Q3) Quartiles. Line inside box = Median.

When comparing USP14 expression levels between patients who did and did not recur within 36 months, the median USP14 expression level was higher among the recurrent cases (Figure 1; Median [Range]: 2.3 [1.7-3.0] vs. 2.0 [1.0-3.0], respectively; p=0.02). In order to address whether this association was independent of other risk factors of recurrence, a multivariate logistic regression model was constructed to adjust for grade, disease stage, lymphovascular space invasion, histology and receipt of adjuvant therapy. After adjustment, higher USP14 expression levels were highly associated with recurrence (odds ratio = 7.6 [95% confidence interval: 1.6-35.3], p=0.01; Table 2).

**Table 2:** Multivariate logistic regression model of risk factors for recurrence within 36 months of diagnosis with stage I endometrial adenocarcinoma

Variable	Odds ratio (95% CI)	p-value
**USP14 grade (per increase in USP14 staining grade)**	7.6 (1.6, 35.3)	0.01
**Grade**		0.27
1	1.0	
2	1.2 (0.2, 6.2)	
3	4.8 (0.6, 35.3)	
**Stage**		0.01
IA	1.0	
IB	6.2 (1.5, 25.5)	
**Lymphovascular Space Invasion**		0.23
No	1.0	
Yes	2.7 (0.5, 13.2)	
**Adjuvant Therapy**		0.32
No	1.0	
Yes	0.5 (0.1, 2.0)	
**Histology**		0.31
Endometrioid	1.00	
Non-Endometrioid	2.8 (0.4, 20.6)	

**Figure 2:**
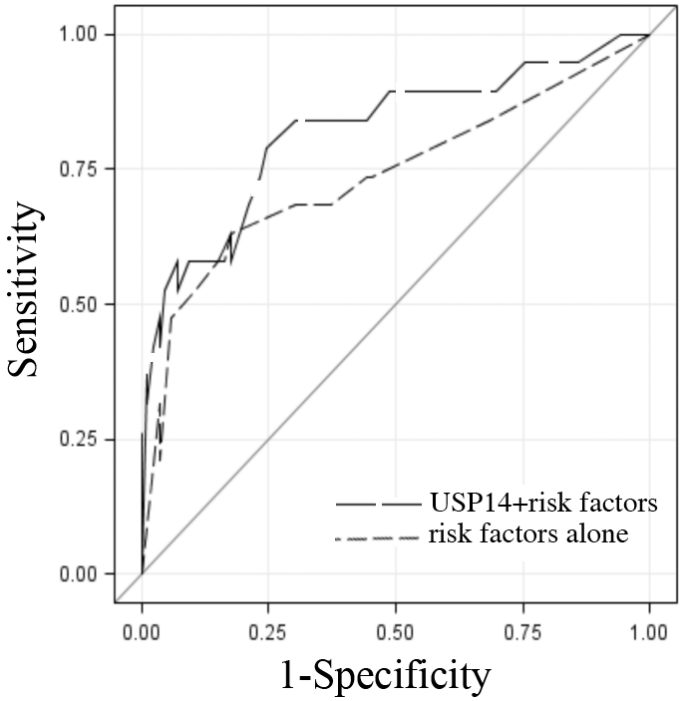
USP14 staining intensity in addition to known predictors of recurrence Receiver operating characteristic (ROC) curves of USP14 staining intensity and risk factors of recurrence (stage, grade, histology, lymphovascular space invasion) (*black long dashed line*) and the risk factors alone (*gray short dashed line*) indicating their ability to differentiate patients with endometrial adenocarcinoma by recurrence status. The solid diagonal line indicates no predictive value. Area under the ROC curve estimates and 95% confidence intervals were 0.82 [95% CI: 0.70-0.94] and 0.74 [95% CI: 0.59-0.89], respectively (p=0.05).

Further, to explore the potential clinical utility of USP14 expression levels, the predictive ability of USP14 when added to known risk factors (grade, disease stage, lymphovascular space invasion and histology) was assessed. The combination of USP14 expression level and these risk factors was superior to the risk factors alone in predicting recurrence, with area under the curve estimates and 95% confidence intervals of 0.82 [95% CI: 0.70-0.94] and 0.74 [95% CI: 0.59-0.89], respectively (p=0.05; Figure 2).

### USP14 is aberrantly expressed in highly proliferating endometrial cancer cells *in situ*

**Figure 3:**
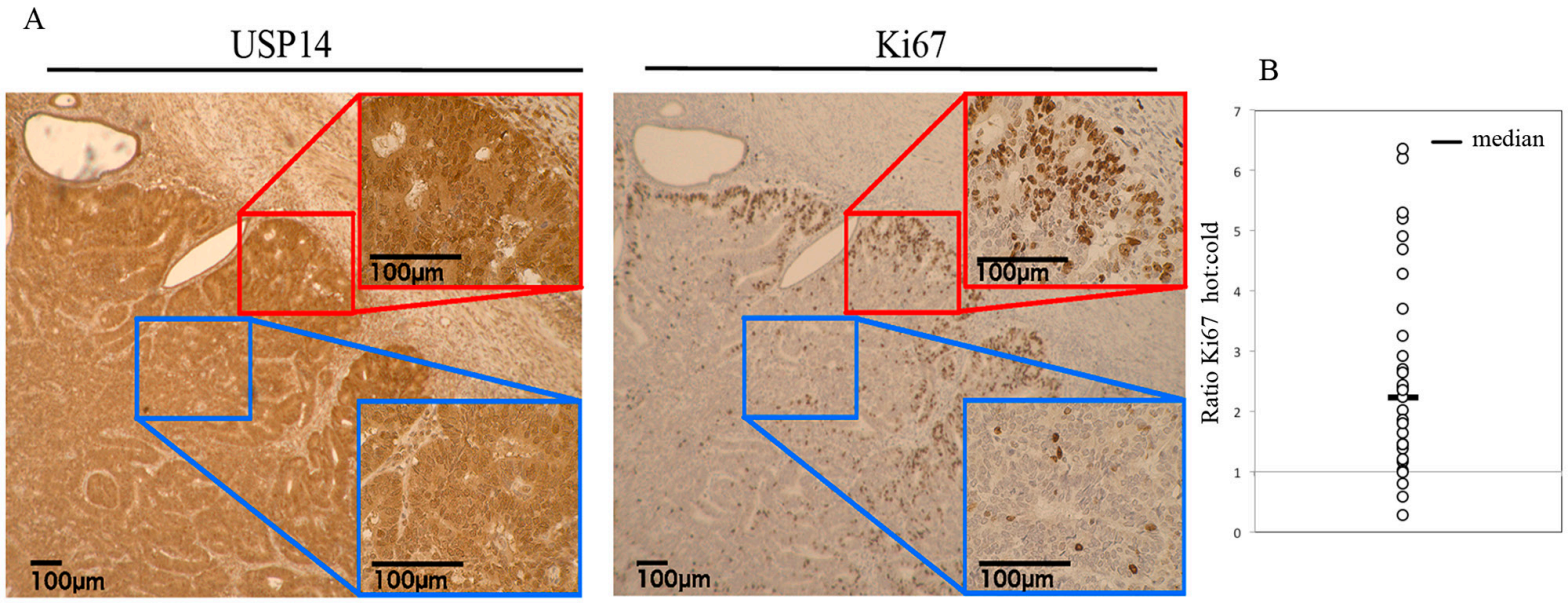
USP14 is overexpressed in proliferating endometrial cancer cells in situ **A.** Immunohistochemical staining of USP14 and Ki67 in endometrial cancer tumors. Representative examples of strong (*red window/hot*) and weak (*blue window/cold*) USP14 (left panel) and Ki67 (right panel) staining intensity, respectively. **B.** Dot plot of the ratio of the number of Ki67 stained cells in areas with strong USP14 expression (hot) to areas with weak USP14 expression (cold) in 31 endometrial cancer tumor samples. Line = Median.

Uncontrolled proliferation is a key characteristic of cancer and therefore is strongly associated with prognosis [17]. Ki67 is a marker of proliferation that has been used extensively in cancer research, particularly breast cancer, and has been shown to be associated with outcomes [18]. To examine whether USP14 expression levels were elevated among highly proliferative cells, we assessed the relationship between USP14 and Ki67 in a subset of 31 patient samples. Specifically, the number of Ki67 stained cells in corresponding areas of USP14 weak (low intensity) and strong (high intensity) fields for each patient were determined. More than twice as many Ki67 stained cells in the strong USP14 staining intensity areas were found compared to the weak USP14 staining intensity areas (ratio=2.2, p<0.0001; Figure 3). This suggests increased USP14 expression levels could be either a requirement for or a consequence of cancer cells' proliferation and of tumor aggressiveness *in vivo*.

### Inhibition of USP14 results in decreased cell viability in carboplatin resistant endometrial cancer cell lines

**Figure 4:**
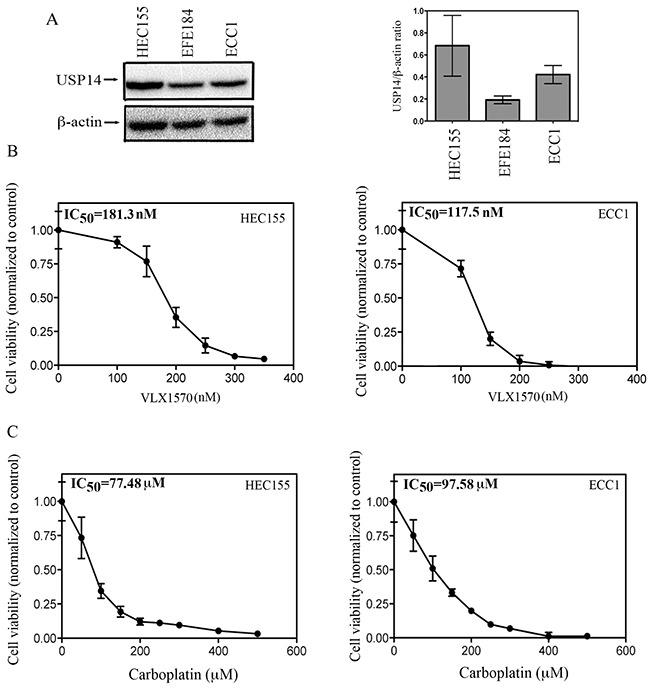
Effect of USP14 inhibition on carboplatin resistant endometrial cancer cells **A.** (left panel), Western blot analysis for USP14 expression levels in the endometrial cancer cell lines HEC155, EFE184 and ECC1. Equal protein loading in each line was verified using an antibody against β-actin. Right panel, quantification of USP14 expression in endometrial cancer cell lines expressed as USP14/ β-actin ratio. Three independent experiments; means ± standard deviations are presented. **B.** dose-dependent inhibition of cell viability of HEC155 (left panel) and ECC1 (right panel) endometrial cancer cell lines exposed to increasing concentrations of VLX1570 over a period of 48 hours. Percentage of viable cells is relative to mock-treated controls. Three independent experiments performed in triplicate; means ± standard deviations are presented. **C.** dose-dependent inhibition of cell viability of HEC155 (left panel) and ECC1 (right panel) endometrial cancer cell lines exposed to increasing concentrations of carboplatin over a period of 48 hours. Percentage of viable cells is relative to mock-treated controls. Three independent experiments performed in triplicate; means ± standard deviations are presented.

We next wanted to investigate whether USP14 is a molecular target for endometrial cancer cells. To this end, we first measured the expression levels of USP14 in a panel of endometrial cancer cell lines including HEC155, EFE184 and ECC1. These cell lines were chosen as they represent the most aggressive and most likely to recur endometrial cancer types [7, 19]. As shown in Figure 4A (left panel), all cell lines tested expressed USP14, with HEC155 and ECC1 having the highest levels. Quantification of the USP14 expression levels, expressed as the ratio to β-actin is shown in Figure 4A (right panel). Next, ECC1 and HEC155 endometrial cancer cells were exposed to increasing concentrations of the FDA approved small-molecule inhibitor VLX1750 and the residual cell viability was measured after a period of 48 hours. As shown in Figure 4B, pharmacological inhibition of USP14 caused dose-dependent inhibition of cell viability in endometrial cancer cell lines with an IC_50_ of 181.3 and 117.5 nM for HEC155 (left panel) and ECC1 (right panel), respectively.

In the clinical setting, recurrent endometrial cancer is usually resistant to chemotherapy. Previous studies have reported that among endometrial cancer cell lines, HEC155 and ECC1 have the highest levels of L1CAM, a marker for endometrial cancer recurrence [7, 19]. This suggests that these cell lines are appropriate *in vitro* models for more aggressive endometrial cancer. This is consistent with earlier reports indicating that the ECC1 cell line has a high degree of resistance to both cisplatin and carboplatin *in vitro* [20]. Thus, we measured the IC_50_ levels for carboplatin treatment of HEC155 and ECC1 cells. As shown in Figure 4C, the IC_50_ levels for carboplatin were 77.5 and 97.6 μM for HEC155 (left panel) and ECC1 (right panel) respectively. Taken together this suggests a concentration-dependent anti-proliferative effect of the USP14 inhibitor VLX1570 in endometrial cancer cells at concentrations that are 500 and 800 times lower than that required for carboplatin.

### Treatment of endometrial cancer cells with a small-molecule proteasome-associated inhibitor severely compromises ubiquitin-dependent protein degradation

**Figure 5:**
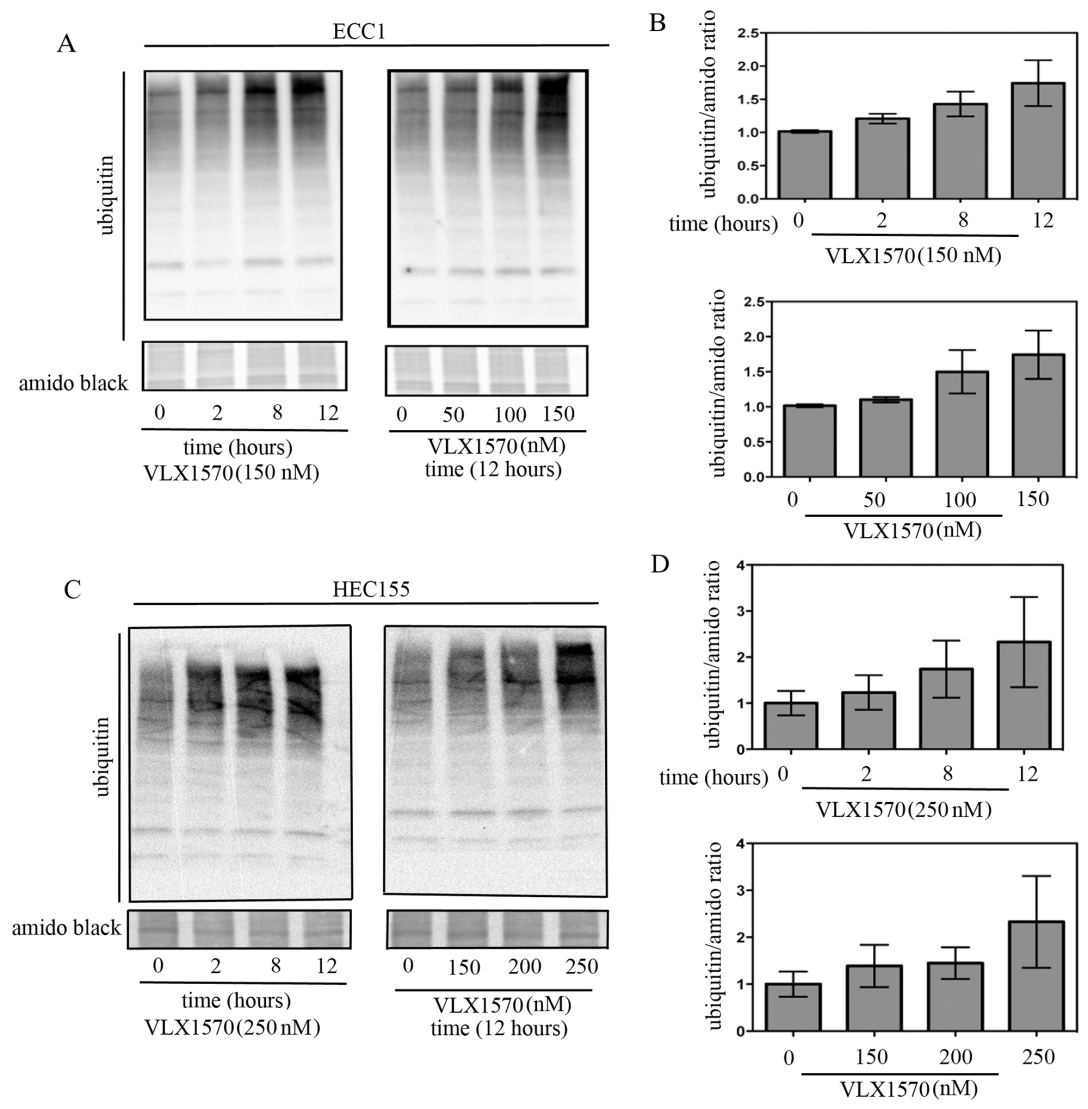
Time and dose-dependent effects of USP14 inhibition on degradation of ubiquitinated proteins **A.** Time- (left panel) and dose-dependent (right panel) effects of VLX1570 treatment on the accumulation of poly-ubiquitinated proteins in the ECC1 endometrial cancer cell line. **B.** Quantification of the ubiquitin/amido black ratios for time- (top panel) and dose-dependent (bottom panel) VLX1570 treatment. Three independent experiments; means ± standard deviations are presented. **C.** Time- (left panel) and dose-dependent (right panel) effects of VLX1570 treatment on the accumulation of poly-ubiquitinated proteins in the HEC155 endometrial cancer cell line. **D.** Quantification of the ubiquitin/amido black ratios for time- (top panel) and dose-dependent (bottom panel) VLX1570 treatment. Three independent experiments; means ± standard deviations are presented.

Along with UCHL5, USP14 is a proteasome-associated DUB whose role is to remove ubiquitin molecules from targeted proteins prior to degradation by the 20S catalytic activities of the proteasome. Aggressive endometrial cancer cells express abnormally high levels of USP14 *in situ* suggesting a higher requirement for proteasome-associated DUB activity. Thus, we investigated the consequences of inhibition of proteasome-associated DUBs with VLX1570 on ubiquitin-dependent protein degradation in endometrial cancer cells. To this end, ECC1 and HEC155 cells were exposed to 150 nM or 250 nM VLX1570, respectively, over a period of 12 hours and the effect on cellular protein ubiquitination was evaluated by Western blot analysis after 0, 2, 8 or 12 hours from drug exposure. As shown in Figure 5A and 5C (left panels), VLX1570 treatment resulted in a dose-dependent accumulation of poly-ubiquitinated proteins in ECC1 and HEC155 cell lines starting as early as two hours following drug exposure. Quantifications of the changes in high molecular weight ubiquitin species in each respective cell line, versus control, are given in Figure 5B and 5D (top panels).

Next, endometrial cancer cell lines were exposed to increasing concentrations of VLX1570 (0–150 or 0-250 μM for ECC1 and HEC155, respectively) over a period of 12 hours. The effect on accumulation of poly-ubiquitinated proteins was evaluated by Western blot. As shown in Figure 5A and 5C (right panels), drug treatment resulted in a dose-dependent inhibition of ubiquitin-dependent protein degradation in endometrial cancer cells. Quantifications of the changes in high-molecular weight ubiquitin species in dose-dependent fashion are given in Figure 5B and 5D (bottom panels). Taken together, these data suggest that the loss of cell viability following proteasome-associated DUB inhibition may be explained by the inability of endometrial cancer cells to cope with increasing levels of proteotoxic stress.

### USP14 inhibition induces G2-M cell cycle arrest and caspase-mediated apoptosis in endometrial cancer cells

**Figure 6:**
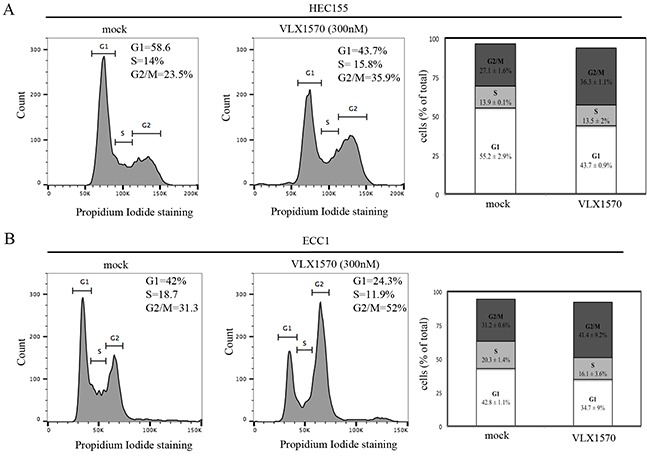
USP14 inhibition induces G2-M cell cycle arrest in endometrial cancer cells **A.** HEC155 endometrial cancer cell line mock (left panel) or VLX1570 treated (middle panel) for 24 hours prior to propidium iodide staining and flow cytometric analysis to determine their cell cycle distribution. Insets correspond to percentage of cells in G1, S, and G2/M phases of the cell cycle. Right panel, graphical representation of cell cycle distribution in control versus VLX1750 treated cells. Results are expressed as % of cells (out of total) in each phase of the cell cycle. Three independent experiments; means ± standard deviations are presented. **B.** EEC1 endometrial cancer cell line mock (left panel) or VLX treated (middle panel) for 24 hours prior to propidium iodide staining and flow cytometric analysis to determine their cell cycle distribution. Insets correspond to percentage of cells in G1, S, and G2/M phases of the cell cycle. Right panel, graphical representation of cell cycle distribution in control versus VLX1750 treated cells. Results are expressed as % of cells (out of total) in each phase of the cell cycle. Three independent experiments; means ± standard deviations are presented.

It has been shown that USP14 modulates levels of key cell cycle regulatory proteins whose dysregulation is expected to affect the cell cycle [21]. This is further supported by our findings of a strong correlation between USP14 and Ki67 staining in clinical specimens of endometrial cancer. Thus, we tested the hypothesis that inhibition of USP14 would result in endometrial cancer cells failing to progress through the cell cycle. First, HEC155 and ECC1 endometrial cancer cells were incubated with the USP14 inhibitor VLX1570 over a period of 24 hours and the cell cycle status was analyzed by flow cytometry after staining with propidium iodide. We found treatment with 300 nM VLX1570 resulted in a shift in the cell cycle distribution in both endometrial cancer cell lines tested. Specifically, treatment of both HEC155 and ECC1 cells led to an increase in the percentage of cells in the G2/M phase of the cell cycle as compared to controls (Figure 6A and 6B). These results suggest that blocking USP14 activity impedes the cells progression through the cell cycle, arresting them in the G2/M phase.

**Figure 7:**
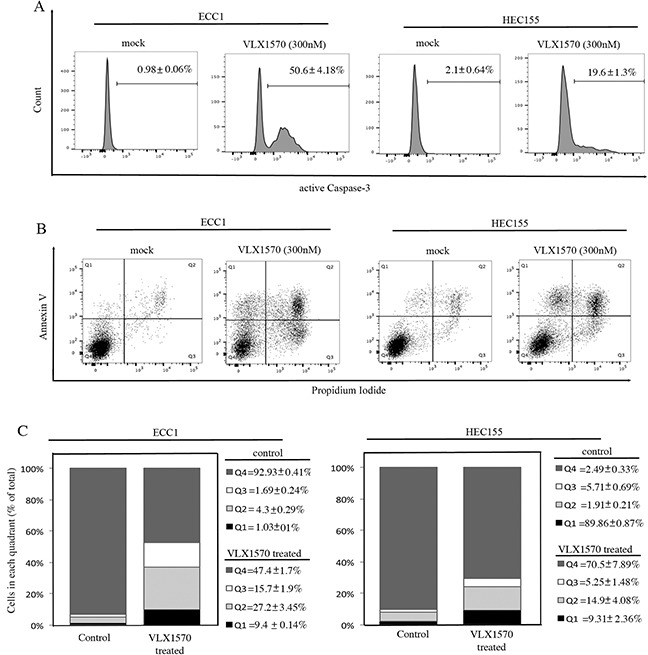
Reduction of cell viability in endometrial cancer cell lines exposed to VLX1570 is consistent with apoptosis-mediated cell death **A.** ECC1 (left panel) and HEC155 (right panel) endometrial cancer cells were mock or VLX1570 treated over a period of 24 hours. The cells were harvested, fixed and stained for the active form of Caspase-3. Three independent experiments; the mean percentage of cells within each gate ± standard deviation is presented. **B.** ECC1 (left panel) and HEC155 (right panel) endometrial cancer cells were mock or VLX1570 treated over a period of 24 hours. The cells were harvested and stained for DNA content (propidium iodide) and Annexin-V. The percentage of cells within each gate is indicated. The quadrants represent living cells (Q4), early apoptosis (Q3), late apoptosis (Q2) and necrosis (Q1). **C.** Quantification of the % of cells (out of total) in each quadrant. Three independent experiments; means ± standard deviations are presented.

Next, we evaluated the fate of ECC1 and HEC155 endometrial cancer cells following VLX1570-induced cell cycle arrest. We tested whether the reduction in cell viability following drug treatment is consistent with onset of apoptosis. First we measured the expression levels of active caspase-3 in control versus VLX1570 treated cells. Importantly, caspase-3 is an active cell-death protease involved in the execution phase of apoptosis, where cells undergo morphological changes such as DNA fragmentation, chromatin condensation and apoptotic body formation. As shown in Figure 7A, exposure to VLX1570 resulted in increased levels of active caspase-3 in both of the endometrial cancer cell lines tested.

To further confirm this result, VLX1570-exposed ECC1 and HEC155 cells were analyzed by flow cytometry after staining with Annexin V. Annexin V protein specifically binds phosphatidylserine, a phospholipid normally localized on the inner leaflet of the plasma membrane which flips to the outer leaflet during early apoptotic signaling [21]. As shown in Figure 7B, treatment with 300nM VLX1570 for 24 hours resulted in an increase of Annexin V staining cells in control versus treated cells. Similarly, the fraction of cells taking up propidium iodide, a feature of both advanced apoptosis and necrosis, is elevated in controls versus treated cells. Quantification of Annexin positive cells per each condition is given in Figure 7C.

These findings support our hypothesis that pharmacological inhibition of USP14 triggers apoptosis in endometrial cancer cells.

## DISCUSSION

While most women with early stage endometrial adenocarcinoma have a favorable prognosis, a subset unexpectedly experience recurrence. In the current study, over 70% of women in our population who recurred had endometrioid histology. This highlights the challenge in the management of early stage low-risk endometrial cancer and suggests a role for clinically applicable prognostic markers. We found that higher USP14 expression levels were independently associated with recurrence and importantly added predictive value after accounting for known risk factors. This suggests a role for USP14 in risk stratification for recurrence and may have implications for clinical care. Further validation in a larger external dataset is needed, however. Another marker, L1CAM, has been found to be a negative prognostic marker for Type I, stage I endometrial cancer and predicts need for adjuvant therapy [7]. It is likely that accurate prognosis will necessitate the use of multiple markers.

Importantly, USP14 may also serve as a potential therapeutic target. Our data show a strong positive correlation between the intensity of USP14 staining and degree of proliferation as measured by Ki67 staining in clinical specimens of endometrial cancer *in situ*. This, along with previously published reports showing that USP14 expression levels fluctuate within cancer cells as they progress through the cell cycle [12], suggests that highly proliferating cells may have greater dependence on USP14 activity. Further, our data showed that pharmacological inhibition of USP14 with the FDA approved inhibitor VLX1570 [13, 22–26] was accompanied by a reduction of the cell viability of endometrial cancer cells with resistance to carboplatin. This is consistent with the knowledge that inhibition of the ubiquitin-dependent protein degradation pathway upstream of 20S proteasome has been shown to reverse chemoresistance to DNA damaging agents as well as 20S proteasome inhibitors in a number of cancer settings, possibly via restoring expression levels of pro-apoptotic proteins including MCL1 [27–29].

Cell cycle is a tightly regulated event under the control of numerous cyclins, cyclin-dependent kinases, and checkpoint proteins which are client proteins of the ubiquitin-proteasome-system. Specifically, pharmacological inhibition of USP14 as well as its genetic silencing has been shown to result in accumulation of high-molecular weight client proteins including cyclin A and B, suggesting that USP14 activity may be required to regulate their steady-state levels [30–32]. This is consistent with our data indicating that reduction in cell viability following VLX1570 exposure is preceded by accumulation of the cells in G2-M. This is also consistent with activation of caspase-3 mediated apoptosis following VLX1570 exposure as a consequence of the inability of endometrial cancer cells to progress through anaphase [33].

While these results are encouraging, this study is not without limitations. The retrospective nature of the patient data may lead to biases due to patient selection, reliance on the electronic medical record, and lack of standardization in surgical and treatment methods. This was an exploratory analysis and therefore women with all types of stage I disease were included, regardless of histology or other known risk factors. In addition, the sample size was relatively small so subgroup analyses were not possible. Prospective studies are needed to validate our findings and to determine the clinical utility of USP14.

Collectively, our experiments support the notion that USP14 shows promise as a potential biomarker for recurrent disease and that inhibition of USP14 may be of therapeutic benefit for women with endometrial adenocarcinoma.

## MATERIALS AND METHODS

### Patient samples

Approval for this study was granted by the University of Minnesota Institutional Review Board. Patients diagnosed with endometrial adenocarcinoma between January 2000 and July 2012 were identified by querying the gynecologic cancer database at the University of Minnesota. Inclusion criteria were as follows: 1) surgical staging including total hysterectomy and bilateral salpingo-oophorectomy with or without pelvic and periaortic lymph node dissection and omentectomy; 2) histologically confirmed endometrial adenocarcinoma; 3) confirmed stage I disease, retrospectively determined according to the FIGO 2009 criteria [34]; and 4) minimum of 36 months follow-up data available in the medical record. Patient demographic and clinical data were extracted from the electronic medical record, including age at diagnosis, race, body mass index, parity, menopausal status, medical comorbidities, disease stage, adjuvant therapy received, and dates of recurrence and death. The pathologic diagnosis was confirmed in each case by a board-certified pathologist. Histologic characteristics including tumor grade, histologic subtype, lymphovascular space invasion, maximum myometrial invasion, and other pathologic characteristics were also recorded.

A total of 203 patients diagnosed with stage I endometrial adenocarcinoma met the inclusion criteria. Of those, a representative sample of 107 were selected for staining with an antibody for USP14, oversampling those who experienced a recurrence. A representative block of tumor was selected from each case by the pathologist. A five micron thick unstained section was cut from each block and mounted onto a glass slide. The unstained slides were subjected to immunohistochemstry against USP14 and Ki67.

### Immunohistochemistry for USP14 and Ki67

Five-micron thick formalin-fixed, paraffin-embedded sections were deparaffinized and rehydrated by sequential washing with xylene, 100% ethanol, 95% ethanol, 80% ethanol, and PBS. For antigen retrieval, slides were immersed in Reveal Decloaker (Biocare Medical, Concord, CA) and steamed for 30 min at 100 degrees C. Endogenous peroxidase activity was blocked with 3% H_2_O_2_ for 10 min. After washing with PBS, slides were blocked with 10% normal goat serum in PBS for 10 min at room temperature, followed by incubation with rabbit anti-human polyclonal USP14 antibody (Bethyl Laboratories) at a concentration of 1:750 in blocking solution overnight at 4 degrees C. After washing twice with PBS, slides were incubated with a biotinylated anti-rabbit secondary antibody conjugated (10 min) and streptavidin/horseradish peroxidase (10 min; Dako), followed by 3,3-diaminobenzidine (Phoenix Biotechnologies) substrate for 3 min. Slides were lightly counterstained with Gill No. 3 hematoxylin (Sigma) for 60 s, dehydrated, and coverslipped.

All of the USP14 immunostained slides were reviewed by two independent pathologists in addition to a panel of five basic scientists, resulting in three measures for each slide. All reviewers were blinded to the clinical outcome of the corresponding patients. The staining intensity was rated as follows: 0 = no staining, 1+ = weak intensity, 2+ = moderate intensity and 3+ = high intensity.

A subset of patients tumor samples (n=31) were additionally stained for Ki67 using the same procedure as described above with the substitution of the Ki-67 monoclonal mouse antibody MIB-1 (Dako, Carpinteria, CA) as the primary antibody at a concentration of 1:150. The correlation between USP14 and Ki67 was evaluated by two independent researchers. Blind to the Ki67 staining, each researcher chose one field each per patient that represented low and high intensity USP14 staining areas, respectively. They then identified the corresponding area of Ki67 staining and counted the number of Ki67 stained cells in one high power field (40x) in the areas corresponding to USP14 weak (blue window/cold) and strong (red window/hot) intensity.

### Chemicals

The USP14 inhibitor VLX1570 was synthetized as previously described [22]. The 2,3-bis[2-methoxy-4-nitro-5-sulfophenyl]-2H-tetrazolium-5-carboxanilide inner salt (WST-1) was purchased from Cayman Chemicals. Propidium iodide was purchased from Sigma.

### Cell lines

The endometrial cancer cell lines were obtained from the following sources: ECC-1 (American Type Culture Collection); EFE-184 (German Tissue Repository DSMZ); HEC-155 (Japanese Health Science Research Bank). Cell lines were cultured in DMEM supplemented with 10% fetal bovine serum, 100 IU/mL penicillin, and 100 μg/mL streptomycin at 5% CO_2_.

### Cell viability assay

Cell viability was determined by 2,3-bis[2-methoxy-4-nitro-5-sulfophenyl]-2H-tetrazolium-5-carboxanilide inner salt assay as previously described [35, 36]. Briefly, cells were seeded at the concentration of 1,000 per well in 100 mL medium in 96-well plate and treated with the indicated concentrations of drugs. At the indicated time points, cells were incubated according to the manufacturer's protocol with the WST-1 labeling mixture for 2 hours. Formazan dye was quantified using a spectrophotometric plate reader to measure the absorbance at 450nm (ELISA reader 190; Molecular Devices). Each experiment was performed in triplicate.

### Antibodies and western blot analysis

Total cellular protein (10–20 μg) from each sample was separated by SDS-PAGE, transferred to PVDF membranes and subjected to Western blot analysis. Antibodies for Western blot analysis were obtained by the following commercial sources: anti-USP14 (Bethyl Laboratories), anti-ubiquitin (Santa Cruz Biotechnology), anti-β-actin (Sigma). Peroxidase-linked anti-mouse Immunoglobulin G and peroxidase-linked anti-rabbit Immunoglobulin G were from Amersham. Each experiment was performed in triplicate.

### Antibodies and flow cytometry

Cell cycle status following treatment with drug or vehicle alone was determined via flow cytometry analysis. Specifically, cells were harvested at indicated time points and fixed in 70% ethanol on ice for 2 hours. Following washing with PBS, cells were stained with 0.1% (m/v) propidium iodide in PBS-T. Apoptosis was measured using antibodies against active Caspase-3 (BD Pharmingen) or Annexin V (BD Pharmingen) which were combined with propidium iodide staining (Sigma-Aldrich). For apoptosis assays, samples were fixed and stained according to manufacturer's instructions. Fluorescence was measured with a FACSCantoII flowcytometer (Becton Dickinson) and analyzed with FlowJo software. Each experiment was performed in triplicate.

### Statistical analysis

The goal of the analysis was to determine the association between USP14 staining intensity and recurrence of endometrial adenocarcinoma within 36 months of diagnosis among women with stage I disease. The mean USP14 straining level of the three values (two pathologists and panel of scientists) was calculated for each patient and used for analyses. Available patient demographic and clinical data were summarized and compared by recurrence status using Chi-squared and Fisher's Exact tests. Similarly, USP14 staining intensities were compared across demographic and clinical variables to identify potential confounding factors. A multivariate logistic regression model was conducted to determine the additional utility of USP14 staining intensity as a predictor of recurrence, given knowledge of other risk factors including age, obesity, histology (endometrioid/other), highest pathology grade, disease stage (IA/IB), lymphovascular space invasion (yes/no) and adjuvant therapy received (yes/no). Due to the small sample size, the final multivariate model was selected using backwards selection, keeping variables with p<0.10 or those previously known to be predictive of recurrence such as histology. To explore the predictive value of USP14, a receiver operating characteristic (ROC) curve analysis was conducted, comparing the addition of USP14 to stage, grade, lymphovascular space invasion and histology as compared to those risk factors alone. The area under the ROC curve (AUC) estimates and 95% confidence interval are presented and were compared [37]. Finally, the difference in the median number of USP14 stained cells between the strong and weak Ki67 areas, using the mean value of the two researchers, was analyzed using the Wilcoxon Signed Rank Test. All analyses were conducted using SAS version 9.3 (Cary, NC) and p-values <0.05 were considered statistically significant.
